# Blood Management in Revision Total Hip Arthroplasty for Metal-on-Metal Devices: The Efficiency of an Intraoperative Cell Salvage System

**DOI:** 10.1007/s43465-019-00026-0

**Published:** 2020-01-24

**Authors:** Werner Maurer-Ertl, Andreas Fellner, Patrick Reinbacher, Michael Maier, Andreas Leithner, Joerg Friesenbichler

**Affiliations:** 1 Department of Orthopaedics and Trauma, Medical University of Graz, Auenbruggerplatz 5, 8036 Graz, Austria; 2 Department of Anaesthesiology, Medical University of Graz, Auenbruggerplatz 5, 8036 Graz, Austria

**Keywords:** Arthroplasty, Hip, Cobalt, Chromium, Blood salvage

## Abstract

**Background:**

The aim of this series was to investigate the efficiency of an intraoperative cell salvage system (ICS) removing metal ions during revision of metal-on-metal (MoM) devices to proof the possibility of re-transfusion of the collected blood.

**Materials and methods:**

Between 2014 and 2018, five consecutive patients underwent revision surgery of their MoM total hip arthroplasty due to wear of the polyethylene-metal sandwich inlay or local massive metallosis with aseptic loosing of the cup. Aspiration of joint fluid of all hip prostheses was done and blood was taken to measure the metal ion concentrations, preoperatively. Perioperatively, blood was collected using an ICS before and after filtration and analyzed for Co and Cr concentrations. At that time, there was no re-transfusion of the collected and filtrated blood due to unknown metal ion concentrations.

**Results:**

The mean preoperative serum Co and Cr concentrations in the blood were 31.28 μg/L (range 0.22–77.47) and 17.33 μg/L (range 0.59–51.31), whereas the mean local concentrations in the aspiration fluid were 728-fold and 822-fold higher. The Co and Cr concentrations measured in the collected blood before filtration were 70.61 μg/L (range 9.40–173.00) and 337.21 μg/L (range 8.76–1383.0) and decreased markedly to average concentrations of 15.49 μg/L and 41.88 μg/L, respectively. These differences were statistically not significant (Co: *p* = 0.117, Cr: *p* = 0.175), although the mean reduction rates were 78% and 88% for Co and Cr, respectively.

**Conclusion:**

The current series showed that in case of revision of MoM hip devices, metal ions are still contained in the collected blood following filtration using a modern high-level ICS. Therefore, we would only recommend blood re-transfusion in case of low preoperative Co and Cr concentrations and sufficient renal function to warrant patients’ safety.

## Introduction

Data of several national and international registries are showing increasing numbers of total hip and knee arthroplasties (THA & TKA) all over the world. On the other hand, the number of revisions for failed arthroplasties is also increasing. The fourth generation of metal-on-metal (MoM) THA was introduced at the beginning of the twenty-first century and was popular for joint replacement due to propagated favorable wear patterns and high range of motion, especially in young and physically active patients. Nevertheless, the number of revisions for failed MoM hip arthroplasties has increased within the last 10 years due to increased metal ion concentrations (Co & Cr) and adverse reactions to metal debris (ARMD), although the long-term effects of systemic exposure still remain unclear [1, 2]. The international accepted threshold of 7.0 μg/L for revision surgery of MoM devices was recommended by the UK Medicines and Healthcare products Regulatory Agency (MHRA) and several orthopedic societies [1, 3].

In the literature, it has been shown that marked blood loss during primary or revision joint arthroplasty may lead to higher rates of transfusion, which may negatively affect surgical outcomes due to possible related complications [4]. Therefore, intraoperative cell salvage (ICS) has been shown to be a valuable and effective method to reduce the requirement of allogenic blood transfusion and costs following THA or TKA [5, 6].

The aim of the current series was to investigate the efficiency of an ICS system removing metal ions during revision of failed MoM devices to perform a re-transfusion of the collected blood. Furthermore, measurements of Co and Cr concentrations were done to investigate if there was a correlation between blood levels, the levels measured in the aspiration fluid, and the levels measured in the ICS system.

## Materials and Methods

Between 2014 and 2018, five consecutive patients underwent revision surgery of their MoM total hip arthroplasty due to wear of the polyethylene-metal sandwich inlay or local massive metallosis with aseptic loosing of the cup following an average follow-up of 172 months (range 85–216) after index procedure. There were four female patients and one male patient. The mean age at time of index surgery was 45 years (range 20–71) and 59 years at time of revision (range 37–88). Patient’s demographics are shown in Table 1.

**Table 1:** Patients’ demographics

No	Sex	Year of implantation MoM device	Type of MoM bearing	Age at index operation (years)	Age at Revision (years)	Time till revision (months)
1	F	2003	Metasul	50	61	143
2	F	1998	Metasul	20	37	212
3	M	1998	Metasul	71	88	205
4	F	2009	ASR XL Head	52	59	85
5	F	2000	Metasul	35	53	216

All hip prostheses underwent percutaneous aspiration to exclude periprosthetic infection as well as to determine local metal ion concentrations; further blood was taken to measure the metal ion levels in the whole blood, 4–6 weeks preoperatively. Perioperatively, blood was collected using an autotransfusion system called OrthoPAT (Haemonetics Corp., Braintree, Mass.) with an integrated percolation system. The SmartSuction Harmony system of the OrthoPAT reduces haemolysis of red blood cells. The wound blood was collected in a filtered reservoir; afterwards, red blood cells were separated by centrifugation and washed with saline solution. The concentrated red blood cells were collected in a transfusion bag. For metal ion determination, blood was obtained from the reservoir bag before and after centrifugation and wash out.

At that time, there was no re-transfusion of the collected and filtrated blood due to unknown metal ion concentrations.

An external laboratory (Medizinische und chemische Labordiagnostik Lorenz & Petek GmbH, Graz, Austria) was hired to determined Co and Cr concentrations. Analysis was done using electrothermal graphite furnace atomic absorption spectrometry (ET ASS). The levels of metal ions were recorded in concentrations expressed as μg/L. Detection limits were 0–0.5 μg/L for Co and 0–1.9 μg/L for Cr.

Statistical analysis was done using the PASW Statistics 22.0 program (SPSS Inc., Chicago, IL). Due to the asymmetric distribution of data, a nonparametric test (Mann–Whitney *U* test) was used. A *p* value of < 0.05 was considered to be statistically significant.

## Results

The mean preoperative whole blood Co and Cr concentrations were 31.28 μg/L (range 0.22–77.40) and 17.33 μg/L (range 0.59–51.31), whereas the local concentrations in the aspiration fluid were 728-fold and 822-fold higher [mean: Co: 22,789.43 μg/L (range 35.10–73,390.00) and Cr: 14,254.71 μg/L (range 4.93–57,500.00), Tables 2 and 3].

**Table 2:** Results of metal ion determination for Cobalt (Co) for each patient in the blood, the aspiration fluid, and in the used intraoperative cell salvage system

No	Whole blood preoperatively	Aspiration fluid	OrthoPAT before filtration	OrthoPAT after filtration
1	0.40	35.10	9.40	0.30
2	71.70	73,390	118	17.00
3	6.70	112.70	43	2.60
4	77.40	40,360	173	57.50
5	0.22	49.34	9.63	0.07
All (mean)	31.28	22,789.43	70.61	15.49

Furthermore, the average results of Co determination for all patients together are used for statistical analysis. All results are shown as μg/L

**Table 3:** Results of chromium (Cr) level determination for each patient in the blood, the aspiration fluid, and in the used intraoperative cell salvage system

No	Whole blood preoperatively	Aspiration fluid	OrthoPAT before filtration	OrthoPAT after filtration
1	0.59	63.10	14.07	1.13
2	51.31	13,560	1383	136.40
3	1.43	4.93	8.76	0.75
4	31.68	57,500	216.50	68.70
5	1.66	145.50	63.70	2.43
All (mean)	17.33	14,254.71	337.21	41.88

Further, average results of Cr determination for all patients together are used for statistical analysis. All results are shown as μg/L

The Co and Cr concentrations measured in the collected blood from the reservoir bag before centrifugation and wash out were 70.61 μg/L (range 9.40–173.00) and 337.21 μg/L (range 8.76–1383.00), respectively. Following centrifugation and wash out, the metal ion levels decreased markedly to an average of 15.49 μg/L (range 0.07–57.50) and 41.88 μg/L (0.75–136.40) for Co and Cr, respectively (Tables 2 and 3). Nevertheless, these differences were statistically not significant (Co: *p* = 0.117, Cr: *p* = 0.175), although the mean reduction rates were 78% and 88% for Co and Cr, respectively. Statistical analysis did not show any significant correlation between measured Co and Cr levels and localisation of determination.

## Discussion

Several studies showed that intraoperative cell salvage reduces allogenic blood transfusion rates in joint arthroplasty and emphasized the cost-effectiveness. These observations are also relevant for revision arthroplasties. The current series showed that in case of revision of MoM bearings, especially older-generations like the Metasul bearing, but also the fourth generation MoM implants, an autologous blood re-transfusion should only be done in selected cases with low local concentrations in the aspiration fluid as well as low preoperative whole blood Co and Cr concentrations to warrant patients’ safety. The recent study clearly showed that metal ions are still contained in the collected blood following centrifugation and wash out, despite using a modern high-level percolated autotransfusion system. Nevertheless, a significant correlation between measured serum metal ion concentrations and metal ion concentrations determined in the aspiration fluid or the blood of the ICS could not be shown. One reason could be the small number of patients enrolled and sometimes differences in measured levels between the various locations might be due to some kind of systemical and local diluting effect.

There are several risk factors for elevated Co and Cr levels following MoM and MoC (metal-on-ceramic) THA reported in the literature. Implant position (inclination), bigger femoral head size, higher range of motion, higher grade of activity, and female sex are known to be at risk for increased metal ion concentrations [2, 7]. Another well-known source of metal ions is the taper junction between the femoral stem and the femoral head [2, 7]. All these factors might also be the cause for the different metal ion levels in the current series.

Sizer et al. [8]. identified preoperative anemia, older age, multiple comorbidities, increased operative time, and postoperative anticoagulation as risk factors for higher blood loss and transfusion rates.

Holt et al. [9]. showed that a multimodal, multidisciplinary approach to perioperative blood management, including preoperative hemoglobin optimization, minimization of perioperative blood loss, and adherence to evidence-based transfusion guidelines, resulted in a significant reduction of transfusion rates in THA and TKA.

Preoperative blood donation and intra- and postoperative blood collection as well as administration of pharmaceutical agents to reduce blood loss (e.g., tranexamic acid) or to stimulate the production of erythrocytes (e.g., erythropoietin) have been proposed as alternative techniques to transfusion of homologous blood [6, 10].

Like in the current series, Ganapathi et al. [11] showed that the processing of recovered blood during revision of MoM devices in a commercial cell saver significantly reduced the total metal load. Furthermore, it was demonstrated that the re-infusion of collected blood did not result in elevated metal ion levels in the immediate postoperative period [11]. In contrast to that, the collected blood in the current study was not re-infused due to the risk of potential side effects or the fear of additional metal ion increment. Furthermore, renal function must always be taken into consideration due to the fact that limited renal function may additionally contribute to an accumulation of metal ions.

In an earlier series, Parker et al. [12] related that leukocyte reduction filters of ICS systems might be effective in reducing metal ion concentrations in recovered blood used for autologous re-infusion, but further investigations were recommended to prove this fact. On the other hand, several authors reported severe cases of neuropathies and intoxications induced by highly increased Co and Cr levels due to excessive wear following metal-on-polyethylene and ceramic-on-metal THA [13–20].

There are several limitations of the current series: (1) there are only five consecutive patients included, but we think that this is enough to show that the usage of a standard ICS is not sufficient for the complete removal of all Co and Cr ions from the salvaged blood; (2) there were no further postoperative metal ion determinations to illustrate the trend of the Co and Cr levels, but, as shown in the literature, we expect a further decline in concentration.

Finally, in cases of revision of MoM devices, we would only recommend the re-transfusion of collected autologous blood in case of low preoperative Co and Cr concentrations and sufficient renal function to warrant patients’ safety. At least, the decision depends on the surgeons’ preference, but the patient has to be informed about possible risks and benefits.
